# Dynamically downscaled 1-km climate dataset for the Balearic Islands under present and future conditions

**DOI:** 10.1038/s41597-026-07301-4

**Published:** 2026-04-30

**Authors:** Yseut Bahuet-Bourret, Tommaso Cancellario, Daniel Argüeso, Enrique Arboleda, Miquel À. Conesa, José M. Escalona, Celso García, María Capa

**Affiliations:** 1 Meteorology Group, Physics Department, University of the Balearic Islands, Palma, Spain; 2 Balearic Biodiversity Centre, Biology Department, University of the Balearic Islands, Palma, Spain; 3 Agro-Environmental and Water Economics Research Institute-University of the Balearic Islands (INAGEA-UIB), Palma, Spain; 4 GLOWATER, Department of Geography, University of the Balearic Islands, Palma, Spain

## Abstract

High-resolution climate data are essential for understanding local climate impacts, assessing vulnerability, managing resources, and developing adaptation strategies in regions sensitive to climate change. This is the case for the Balearic Islands, located in the Western Mediterranean, which are characterized by rich biodiversity, pronounced exposure to global warming, and strong socio-economic dependence on climate-sensitive sectors such as tourism, agriculture, and water resources. We present **Balear1km**, a new climate dataset of dynamically downscaled climate simulations over the Balearic Islands at 1 km spatial resolution and hourly time steps for the period 2009–2023. It includes two simulations produced with the Weather Research and Forecasting (WRF) model: a historical simulation driven by ERA5 reanalysis data, and a future simulation using the Pseudo-Global Warming approach, which applies a climate change signal from 30 global climate models (CMIP6, high-emission scenario SSP5-8.5) to current conditions. This dataset provides physically consistent climate information across land and sea, enabling exploration of how recent weather events may respond under future warming conditions. It can support research and applications in hydrology, ecology, agriculture, public health, and resource management.

## Background & Summary

Growing evidence shows that climate change is already affecting ecosystems and societies^1–3^. As a result, integrating climate information and future projections into land-use planning, biodiversity conservation, and resource-management policies has become an urgent priority^1,4,5^. Access to climate datasets at appropriate spatiotemporal scales is therefore essential to enable this integration. Global Climate Models (GCMs), typically operating at spatial resolutions of hundreds of kilometers, are not designed to capture the intensity of climate extremes, such as intense precipitation events, nor the spatial heterogeneity and microclimatic variation that influence ecological and socio-environmental processes. Regional climate models at intermediate resolutions (~10 km) provide a more detailed representation of these features, but still often lack the granularity required by many end-users or applications. Kilometer-scale climate information at hourly frequency enhances our ability to assess local vulnerability, anticipate future risks and develop effective, place-based adaptation strategies^6,7^.

The Mediterranean is widely regarded as a hotspot for biodiversity^8,9^, as well as for climate change impacts^10–13^. The Balearic Islands, located in the Western Mediterranean, combine unique environmental and socio-economic characteristics^10^, making them a particularly sensitive region^11^ where high-resolution climate models can support a wide range of scientific and practical applications. The region’s economy relies heavily on tourism, which increases pressure on natural resources and ecosystem services^12–14^, and is itself sensitive to climate variability and change. The archipelago also hosts a remarkable number of endemic and threatened species, many of which are highly dependent on local climate conditions^10,15^. In this context, the availability of high-resolution, open-access climate data provides a valuable foundation for interdisciplinary research and evidence-based decision making.

Beyond broad climate indicators, ecology, hydrology, and agriculture often require climate information that resolves the sharp coastal and topographic gradients of islands and keeps the atmosphere–land variables physically consistent. For ecology, climate exerts a strong influence on species occurrence, and kilometer-scale environmental data can help to capture microclimatic heterogeneity that drives species distributions and their susceptibility to environmental change. For hydrology, catchment-scale analyses rely on spatially distributed precipitation and evapotranspiration together with runoff- and storage-related terms, so physically consistent fields at fine spatial resolution help quantify water-balance variability and its sensitivity to climate variability and change^16,17^. For agriculture, Mediterranean production faces increasing pressure from warmer and drier conditions^18,19^, while perennial fruit-tree systems are also sensitive to winter temperature conditions that control dormancy and flowering^20,21^. High-temporal-resolution temperature outputs enable the derivation of temperature-based indices, including chilling metrics, used in agroclimatic assessments^22,23^. By providing paired recent-past and pseudo-future simulations at 1 km and hourly resolution, **Balear1km** supports these communities with a common, physically consistent basis to examine both present-day conditions and fine-scale responses to a climate-change signal.

Previous initiatives have provided climatic information for the Balearic Islands through statistical approaches of varying sophistication. PREGRIDBAL^24^ offers daily precipitation from 1950 to 2009 on a 100-m grid using rain-gauge observations and geostatistical interpolation. Broader regional datasets such as Iberia01^25^ (~11 km) and its predecessor Spain02^26^ (~20 km) also include the archipelago, while E-OBS^27^ delivers continental-scale information over Europe at 0.1° resolution (~11 km at Mediterranean latitudes). These datasets provide valuable climatologies; however, their construction through statistical interpolation of station data makes their accuracy dependent on the density and quality of available observations and limits their ability to resolve local effects of topography, land–sea contrasts, and land use. They generally include only precipitation and/or temperature variables and, by construction, remain restricted to past conditions.

At the global scale, WorldClim v2^28^ and CHELSA^29^ provide widely used high-resolution (~1 km) climate datasets, available at monthly and daily temporal resolution, respectively. Both deliver temperature, precipitation, and derived bioclimatic variables for ecological and environmental analyses. WorldClim statistically interpolates station data using topographic and satellite-observations covariates, improving global temperature patterns but tending to smooth sharp climatic gradients^28^. CHELSA, based on bias-adjusted reanalysis, statistically downscales coarse fields with simplified representations of physical processes, generally improving the depiction of orographic precipitation in several mountainous regions, though with variable performance across locations^30^. The accuracy of both datasets remains constrained by sparse observations and complex terrain: while CHELSA shows marked improvements in some mountain systems, its performance is unverified for the Balearic Islands, where both datasets rely on a very restricted subset of the existing station network. Both initiatives also provide future climate projections derived from global circulation models participating in the Coupled Model Intercomparison Project (CMIP), extending their high-resolution climatologies to future periods under various Shared Socioeconomic Pathway (SSP) scenarios.

To create high-resolution climatic data and overcome the limitations of the aforementioned methods, high-resolution climatic fields can be produced through dynamical downscaling, which explicitly resolves atmospheric processes and are physically consistent. By numerically solving the governing equations of the atmosphere on a finer grid, Regional Climate Models (RCMs) produce physically consistent simulations in which variability, extremes, and feedbacks are explicitly resolved. While statistical methods derive local climate from empirical relationships with predictors such as topography or distance to the coast, dynamical downscaling has been shown to better capture extreme precipitation and fine-scale spatial patterns in regions with complex terrain or convective regimes^31,32^. The main limitation of dynamical downscaling is the large computational cost of resolving processes at all grid points and time steps.

Large national and international projects have undertaken multi-model experiments to produce such datasets, most prominently CORDEX (e.g. www.cordex.org), which coordinates simulations under harmonized protocols across multiple regions. However, typical CORDEX runs at ~12 km still lack the spatial detail required by many applications. To address this, the CORDEX Flagship Pilot Study on Convection (FPS-CONV) coordinated international multi-model experiments at kilometer-scale resolution (~3 km). These simulations at kilometer-scale resolution explicitly resolve convective processes instead of relying on parameterizations, hence the term convection-permitting (CP) simulations, and they consistently improve the representation of sub-daily and extreme precipitation^33–37^. Yet the computational cost further escalates at such resolutions, and the FPS-CONV Mediterranean domain was centered on the Alps, leaving the Balearic Islands at the domain margin, where lateral boundary effects can compromise reliability^38^.

While these efforts have mainly targeted past and present climate, one of our objectives is to assess how small-scale processes may evolve under future conditions. For this, we adopt the Pseudo-Global Warming (PGW) approach, which enables convection-permitting simulations at affordable cost by applying multi-model climate change signals as perturbations to boundary conditions. Unlike separate GCM downscaling^39^, which requires massive computational resources, PGW integrates information from several GCMs into a single experiment, reducing computational demand while avoiding dependence on a single model. However, it is worth noting that this is done at the expense of the uncertainty provided by multiple formal projections.

Here, we present **Balear1** **km**, a dataset that provides dynamically downscaled climate information over the Balearic Islands at unprecedented spatial (1 km) and temporal resolution (hourly). Using the Weather Research and Forecasting (WRF) model, we simulated recent past and future climate conditions across the marine and terrestrial areas of the archipelago. By making these high-resolution model outputs publicly available, we aim to support a broad range of applications such as physical oceanography, biodiversity, land and resource management, hydrology, agriculture, health and climate, among many others.

This dataset emerges from a multidisciplinary initiative at the University of the Balearic Islands to establish a shared climatic framework for research and applied studies across the archipelago. It addresses the lack of physically informed, high-resolution climate information capable of capturing the strong spatial contrasts imposed by the islands’ complex coastal and mountainous landscapes. By providing recent-past and pseudo-future simulations at 1 km resolution, it offers a unique resource for examining both present-day conditions and potential climate-change responses at regional scale.

## Methods

### Methods overview

We dynamically downscale the Copernicus Climate Change Service (C3S) ERA5^40^ reanalysis over the Balearic Islands using the Weather Research and Forecasting (WRF) model v4.6^41^ from a spatial resolution of 0.25° (~31 km) to 1 km for the period 2009–2023, both included. The future climate simulation uses the Pseudo-Global Warming approach, in which a climate change signal derived from 30 GCM datasets from the Coupled Model Intercomparison Project Phase 6^42^ (CMIP6) is added to ERA5 to create perturbed boundary conditions. The historical simulation (Control, CTL) is directly driven by ERA5, while the pseudo-future experiment (Pseudo-Global Warming, PGW) is representative of the climate conditions of the period 2031–2050 under a high-emission scenario (SSP5-8.5). As such, the CTL experiment is aimed at recreating the sequence of weather events observed during the simulated period (2009–2023), while the PGW is designed to investigate how those weather events would respond under climate change conditions. A key strength of this framework is that internal variability is phase-aligned between CTL and PGW, which allows a robust comparison over 15 years periods. By contrast, conventional approaches typically require independent 30-year periods to minimize contamination from phase differences in decadal variability, so the PGW framework reduces computational cost, enabling us to increase the resolution of simulations.

### Input data and acquisition

The historical WRF experiment (CTL) was forced with ERA5 reanalysis fields obtained from the Copernicus Climate Change Service (C3S) Climate Data Store (CDS). We retrieved hourly ERA5 pressure-level variables^43^ (specific humidity *q*, relative humidity *r*, zonal wind *u*, meridional wind *v*, geopotential *z*, temperature *t*) and hourly ERA5 single-level variables^44^ (2-m temperature *t2m*, 10-m winds *u10* and *v10*, surface pressure, mean sea level pressure, and sea surface temperature) for 2009–2023 over a domain enclosing the WRF outer model domain using the CDS web interface. To match the information typically available from CMIP6 GCMs, we also provided WRF with 2-m relative humidity, derived from ERA5 2-m temperature (t2m) and 2-m dewpoint temperature (d2m). For the Pseudo-Global Warming experiment (PGW), the climate change perturbation was computed from a 30-GCM CMIP6 ensemble and added to the ERA5 forcing fields.

CMIP6 monthly model output was obtained from the Earth System Grid Federation (ESGF) archive for the historical and SSP5-8.5 (ssp585) experiments. We used a 30-model CMIP6 multi-model ensemble whose members, institution, experiments and references are listed in Table 1. The CMIP6 ensemble was selected pragmatically for PGW forcing construction: only models providing all required WRF input variables and levels were retained, and one realization per GCM was used to avoid over-representation.

**Table 1:** CMIP6 30-model ensemble used to derive the climate-change signal for the Pseudo-Global-Warming (PGW) experiment.

Model	Institution	Hist	ssp585	Model	Inst	Hist	ssp585
ACCESS-CM2	CSIRO-ARCCSS	CMIP^66^	ScenarioMIP^67^	ACCESS-ESM1-5	CSIRO	CMIP^68^	ScenarioMIP^69^
CanESM5	CCCma	CMIP^70^	ScenarioMIP^71^	CMCC-CM2-SR5	CMCC	CMIP^72^	ScenarioMIP^73^
CMCC-ESM2	CMCC	CMIP^74^	ScenarioMIP^75^	CNRM-CM6-1	CNRM-CERFACS	CMIP^76^	ScenarioMIP^77^
CNRM-CM6-1-HR	CNRM-CERFACS	CMIP^78^	ScenarioMIP^79^	CNRM-ESM2-1	CNRM-CERFACS	CMIP^80^	ScenarioMIP^81^
EC-Earth3	EC-Earth-Consortium	CMIP^82^	ScenarioMIP^83^	EC-Earth3-CC	EC-Earth-Consortium	CMIP^84^	ScenarioMIP^85^
EC-Earth3-Veg	EC-Earth-Consortium	CMIP^86^	ScenarioMIP^87^	EC-Earth3-Veg-LR	EC-Earth-Consortium	CMIP^88^	ScenarioMIP^89^
FGOALS-f3-L	CAS	CMIP^90^	ScenarioMIP^91^	GFDL-ESM4	NOAA-GFDL	CMIP^92^	ScenarioMIP^93^
GISS-E2-1-G	NASA-GISS	CMIP^94^	ScenarioMIP^95^	GISS-E2-1-H	NASA-GISS	CMIP^96^	ScenarioMIP^97^
HadGEM3-GC31-LL	MOHC	CMIP^98^	ScenarioMIP^99^	HadGEM3-GC31-MM	MOHC	CMIP^100^	ScenarioMIP^101^
INM-CM4-8	INM	CMIP^102^	ScenarioMIP^103^	INM-CM5-0	INM	CMIP^104^	ScenarioMIP^105^
IPSL-CM6A-LR	IPSL	CMIP^106^	ScenarioMIP^107^	KACE-1-0-G	NIMS-KMA	CMIP^108^	ScenarioMIP^109^
KIOST-ESM	KIOST	CMIP^110^	ScenarioMIP^111^	MCM-UA-1-0	UA	CMIP^112^	ScenarioMIP^113^
MIROC-ES2L	MIROC	CMIP^114^	ScenarioMIP^115^	MIROC6	MIROC	CMIP^116^	ScenarioMIP^117^
MPI-ESM1-2-HR	MPI-M	CMIP^118^	ScenarioMIP^119^	MPI-ESM1-2-LR	MPI-M	CMIP^120^	ScenarioMIP^121^
MRI-ESM2-0	MRI	CMIP^122^	ScenarioMIP^123^	UKESM1-0-LL	MOHC	CMIP^124^	ScenarioMIP^125^

Historical CMIP and ssp585 ScenarioMIP.

### Pseudo-global warming approach and calculation of the climate change signal

**Fig. 1:**
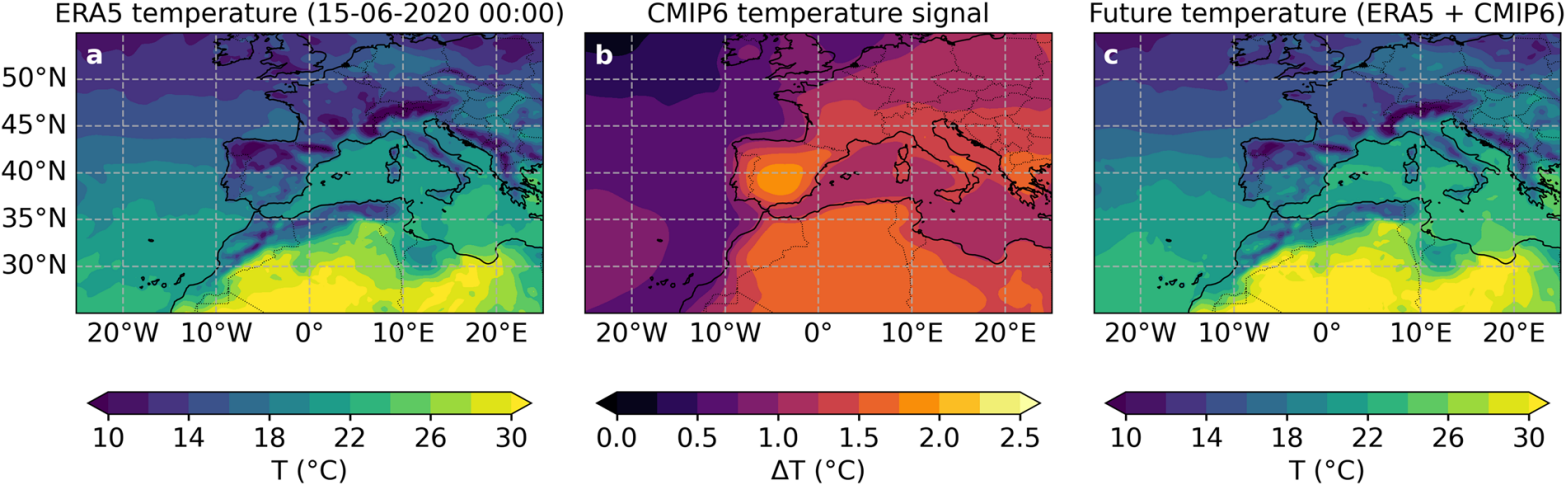
Example of boundary condition construction for the pseudo-future simulation using the Pseudo-Global Warming (PGW) method for 2 m temperature on 15 June 2020 00:00. (**a**) ERA5 2 m temperature field on 15 June 2020 00:00. (**b**) Multi-model mean of CMIP6 GCM monthly (June) 2 m temperature differences between the future (2031–2050) and recent past (2004–2023), calculated as the climate change signal for each model, interpolated to the ERA5 grid. (**c**) PGW boundary condition obtained by summing (**a,b**).

The Pseudo-Global Warming approach^45,46^ used in the future climate experiment (PGW) consists of adding a climate change signal to ERA5. Therefore, the future simulation spans the same years as the historical run (2009–2023) and represents how the atmospheric conditions would have unfolded under a high-level emission scenario. This method – often also referred to as “surrogate warming”^45^, “Thermodynamic Global Warming^47^” and “storylines^48,49^ – vary in complexity, depending primarily on which variables are adjusted and whether the climate change signal is considered constant or time-dependent, with different forms of time dependence further contributing to the methodological diversity. For this dataset, the climate change signal is computed for all variables required by WRF to run both near the surface (2-m temperature, 2-m humidity, 10-m horizontal wind components, surface pressure and mean sea level pressure) and at all vertical levels up to the top of the atmosphere in the model (temperature, humidity, wind components and geopotential). In addition, we perturb the prescribed sea surface temperature (SST) forcing by adding an ocean-surface warming signal derived from CMIP6 surface skin temperature over sea points, while over land, soil variables (temperature and moisture at multiple layers) are computed by the land-surface model and thus incorporate the climate-change signal via the perturbed atmospheric forcing. For each of the variables and each of the 30 GCMs of the CMIP6 experiment, we use monthly data to calculate the climate change signal for each calendar month. The signal is calculated as the difference between the 20-year periods 2031–2050 and 2004–2023. The future period (2031–2050) corresponds to the SSP5-8.5 projections for each GCM, while the recent-past period (2004–2023) combines the historical runs (2004–2014) and the early-century SSP5-8.5 projections (2015–2023), which were concatenated. Once the climate change signal is calculated for each variable, each calendar month and each GCM on their original grid and vertical levels, they were interpolated to the ERA5 grid using nearest-neighbor interpolation to preserve the original information in the GCM and vertically interpolated to ERA5 pressure levels (1, 2, 3, 5, 7, 10, 20, 30, 50, 70, 100, 125, 150, 175, 200, 225, 250, 300, 350, 400, 450, 500, 550, 600, 650, 700, 750, 775, 800, 825, 850, 875, 900, 925, 925, 950, 975 and 1000 hPa). Then all climate change signals are averaged across GCMs to obtain single estimates for each variable and calendar month at every grid point and pressure level on the ERA5 grid. Finally, the averaged climate change signals are linearly interpolated to the 3-hourly frequency of the ERA5 boundary conditions to smooth transitions between calendar months and then added to ERA5 data. As a result, we provide WRF with the sequence of atmospheric conditions corresponding to 2009–2023 as if they would have happened under the global warming projected for 2031–2050 according to a SSP5-8.5 scenario. Figure 1 shows an illustration of how the initial and boundary conditions are constructed for the variable temperature at 2 meters and the datetime 15 June 2020 00:00. This same process is done for each variable, time step, and level. The code to calculate the climate change signal and create the boundary conditions for WRF following this approach is publicly available (see Code Availability section).

### Regional model configuration

**Fig. 2:**
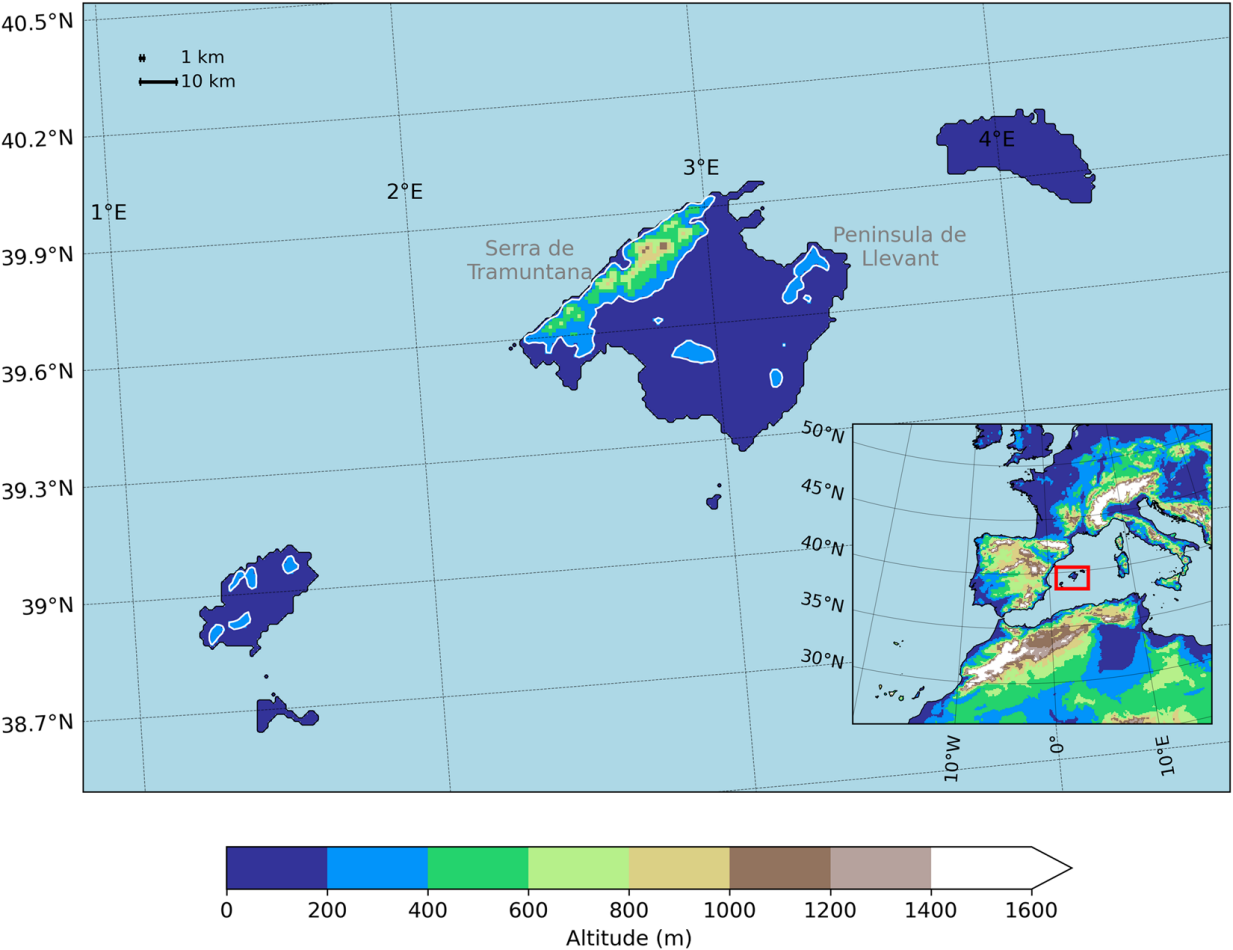
WRF simulation domains with model topography. The main panel displays the child domain at 1 km resolution. The inset shows the parent domain at 10 km resolution, with the red square marking the location of the child domain. The main panel shows 200 m altitude contours in white, highlighting the mountainous areas S*erra de Tramuntana* and *Peninsula de Llevant* in Mallorca Island. From West to East, Ibiza and Formentera Islands, Mallorca, and Menorca.

We used the Weather Research and Forecasting model v4.6^41^ to downscale ERA5 (CTL) and ERA5 plus a climate change signal (PGW) to represent recent-past and future climate conditions. The spatial model configuration consisted of two domains (Fig. 2) to reduce the resolution jump between the boundary conditions and the target domain. The outer domain has a horizontal resolution of 10 km, comprises 361 by 301 grid points, is centered in 40°N 2°W and covers a substantial part of central Europe, northern Africa and the northeast Atlantic Ocean. The inner domain is one-way nested in the outer domain, has a spatial resolution of 1 km, comprises 321 by 221 grid points, is centered in 39.42°N 2.79°W and encompasses the Balearic Islands (Fig. 2). WRF is a non-hydrostatic, fully compressible, mesoscale model used for a wide range of applications including operational weather forecasting, climate projections, and research. It numerically solves the Navier-Stokes equations in their Euler flux form using time-splitting methods (explicit Runge-Kutta scheme for slower processes and a mix of forward-backward and implicit scheme for fast processes) on an Arakawa-C grid with hybrid terrain following vertical coordinates. Like similar models, it provides a wide range of configuration options to represent sub-grid-scale processes known as physical parameterizations (e.g. radiation, turbulence, microphysics). Our choice of parameterization options is based on CORDEX convection-permitting experiments^38,50,51^ and recent studies over the Mediterranean^52,53^.

Our simulations used the WRF single-moment 6-class microphysics scheme, the Rapid Radiative Transfer Model for General Circulation Models for longwave and shortwave radiation, the Yonsei University scheme for turbulence, the Noah Land Surface Model and the Building Effect Parameterization combined with Building Energy Model (BEP + BEM) for the urban environment. Only the outer domain uses Kain-Fritsch parameterized convection, while the deep convective scheme was switched off for the inner domain because its resolution explicitly resolves most convective processes. The sea surface temperature is updated every day, and the land use is described using the CGLC-MODIS-LCZ dataset^54^. The vertical coordinate is resolved with 50 vertical levels, with the default distribution, which ensures higher density near the surface. The bottom level is located approximately 18 m from the surface, and the top level is located at 50 hPa. Each simulation spans 15 years, which were split into 5-year periods, each of them having an additional spin-up period of 6 months that was discarded from the final dataset. The purpose of the spin-up period is to ensure the model reaches an equilibrium between external forcing and internal dynamics, including the adjustment of soil temperature and moisture, which are initialized from an ERA5 climatology and then evolved by the land-surface model. The WRF and WPS configuration (*namelist*) files used to generate simulations are provided in the Supplementary Material to support reproducibility.

## Data Record

The dataset **Balear1** **km** is publicly available through the official repository of the University of the Balearic Islands^55^, hosted on the Dataverse platform and supported by the Consortium of University Services of Catalonia (*Consorci de Serveis Universitaris de Catalunya*). The collection entitled “*Dynamically downscaled high-resolution (1 km) climate information over the Balearic Islands*” can be accessed via the URL https://dataverse.csuc.cat/dataset.xhtml?persistentId=doi:10.34810/data2071.

**Fig. 3:**
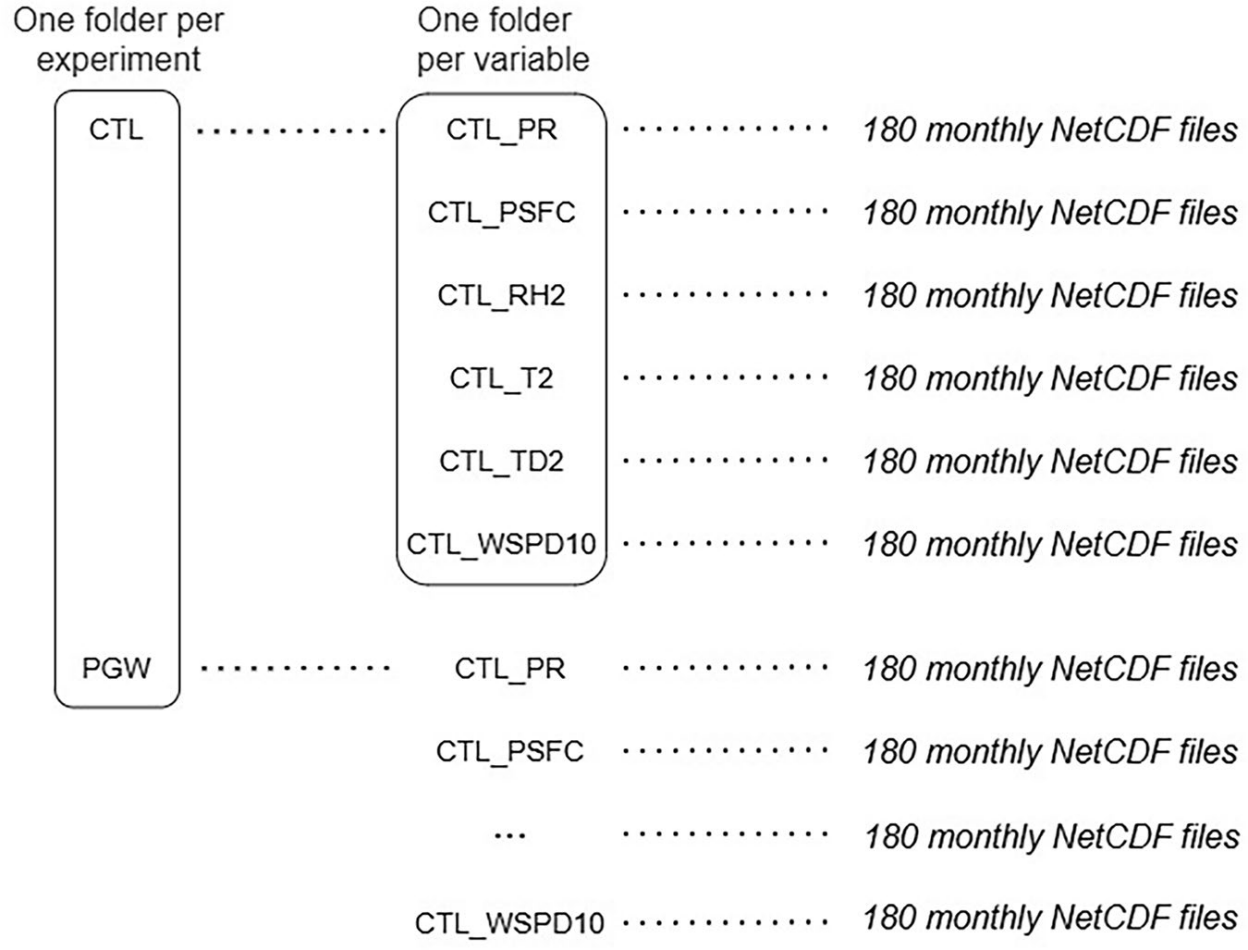
Folder organization of the Balear1km dataset. At the top level, users select between the two experiments (CTL and PWG). Within each experiment folder, subfolders are organized by atmospheric variable. Each variable folder contains 180 NetCDF monthly files for the period 2009–2024, providing hourly data for the full domain.

In total, the dataset contains 1,260 NetCDF files organized in a hierarchical folder structure (Fig. 3). At the first level, users can choose between two experiments: the Control experiment, representing historical conditions for 2009–2023, and the Pseudo-Global Warming experiment, covering the same period but including a climate change signal in the boundary conditions. Within each experiment folder, the atmospheric variables available are precipitation rate (millimeters per hour), surface pressure (Pascals), relative humidity at 2 meters (percent), air temperature at 2 meters (Kelvin), dewpoint temperature at 2 meters (Kelvin), and wind speed at 10 meters (meters per second). Each variable folder contains 180 monthly files for the period 2009–2024, providing hourly values for the entire Balearic Islands domain. Each file name clearly indicates the experiment, variable, and month it represents as the general naming convention is *[EXP]/[EXP]_[VAR]/UIB_01H_[VAR]_YYYY-MM.nc*, where *[EXP]* is the experiment acronym, *[VAR]* the acronym of the atmospheric variable, *YYYY* the four-digit year, and *MM* the two-digit month. The corresponding acronyms used in the dataset directory structure and filenames are listed in Table 2.

**Table 2:** Acronyms used in the Balear1km dataset directory structure and filenames for experiments and atmospheric variables.

Experiments	Description
CTL	Control experiment (historical conditions, 2009–2024)
PGW	Pseudo-Global Warming experiment (same period, with a climate change signal in boundary conditions)

Variables	Description	Units	Height reference
PR	Precipitation rate	mm h^−¹^	–
PSFC	Surface pressure	Pa	–
RH2	Relative humidity	%	2 m
T2	Air temperature	K	2 m
TD2	Dewpoint temperature	K	2 m
WSPD10	Wind speed	m.s^-1^	10 m

The monthly files are post-processed outputs of the Weather Research and Forecasting (WRF) model, and each contains hourly values of a single atmospheric variable over the model domain. This domain has a horizontal resolution of 1 km and is centered at 2.79° longitude and 39.42° latitude. It is composed of 320 grid points in the west–east direction and 220 in the south–north direction, for a total of 70,400 points. In the NetCDF structure, the dimensions x and y are index values that indicate the position of each grid point along these two directions on a grid defined in a Lambert Conformal Conic projection. For practical use, the dataset also provides the latitude and longitude of every grid point, enabling direct comparison of the model output with other geographical data, for example with meteorological station observations.

To facilitate use across disciplines, the dataset follows the Climate and Forecast (CF-1.6) metadata convention, which provides standardized names and units for all variables and attributes. As a result, the files can be directly read and interpreted by a wide range of software tools used in the scientific community.

## Technical Validation

In this Technical Validation section, we evaluate **Balear1km** by comparing model outputs with available observations. Overall, the aim of this validation is to establish Balear1km as a coherent and credible dataset. Because it provides six variables to support a broad range of applications, dedicated fit-for-purpose checks may be needed for specific future uses. The most detailed assessment focuses on over-land conditions, since this dataset was specifically designed to support multidisciplinary studies of climate-change impacts on terrestrial areas across the archipelago; thus, this evaluation highlights the model’s strengths and limitations for end-user applications. At the same time, because the Balearic Islands are an insular region, the simulated climate over the archipelago is strongly influenced by the surrounding marine environment. We therefore include an overview of marine evaluation using buoy observations to check that the model behaves realistically over the ocean. Because of the limitations encountered for precipitation, and given its well-known challenge in regional climate modelling, we additionally benchmark Balear1km precipitation against CHELSA and WorldClim, two widely used high-resolution gridded products that represent the main open-source alternatives currently available for the region. Finally, we assess practical usability, including data access, format, and the availability of spatial/temporal resolution and variables relevant for multidisciplinary applications.

### Marine evaluation using buoy observation

#### Data and preprocessing of marine buoy observation

**Fig. 4:**
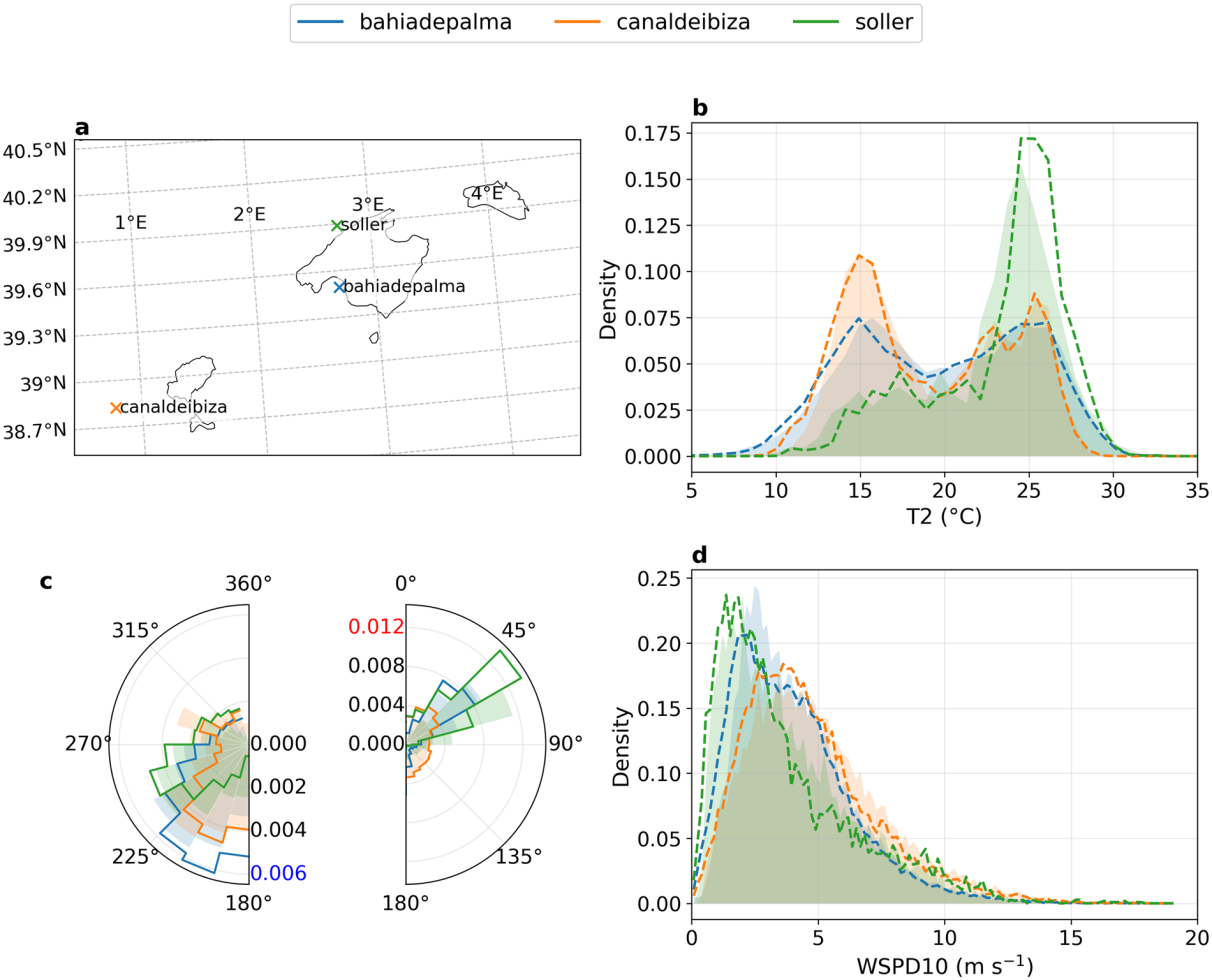
Comparison of SOCIB buoy observations and WRF CTL (present climate) output at the nearest model gridpoint. Shaded areas show the probability density (PDF) of buoy-measured values; dashed lines show the PDF of WRF-simulated values (step lines for wind direction). Panel (**a**) shows the locations of the buoys. Panels (**b**), (**c**) and (**d**) probability density of 2-m air temperature (**b**), 10-m wind direction (**c**) and 10-m wind speed (**d**).

Regarding the offshore validation, we use *in-situ* measurements from fixed mooring buoys provided through the Balearic Islands Coastal Observing and Forecasting System (SOCIB). Within our study region, the SOCIB archive contains usable records for 2009–2023 at three buoy locations (Fig. 4a): *Badia de Palma* and *Sóller* (Mallorca), and *Canal d’Eivissa* (western Ibiza). We applied quality-control of the buoy data using SOCIB QC flags and aggregated to hourly values to match the WRF data. After filtering, the resulting dataset temporal coverage across stations appears site dependent. Badia de Palma provides the longest record with most months covered from 2012–2023, except for a 10-months gap in 2020. Canal d’Eivissa is mostly covering the year 2014–2017. While Sóller is limited to valid data in spring–autumn 2021 and late summer–autumn 2023. We evaluated only paired hours where both the buoy observation and the corresponding WRF value are available.

#### Marine validation of the dataset

The comparison between SOCIB buoy observations and the nearest WRF CTL gridpoint shows overall good agreement over sea for 2-m air temperature, 10-m wind speed, and wind direction (Fig. 4). Note that buoy meteorological sensors are typically mounted only a few meters above the sea surface (often ~1–3 m), which differs substantially from the standard 10 m wind level used in WRF diagnostics and may therefore contribute to discrepancies in the comparison. The 2-m temperature distributions are clearly bimodal at canal de Eivissa and Palma, with two main modes around 15 °C and 25 °C, and the model reproduces both the peak values and their frequency of occurrence. Sóller shows mostly one peak around 25 °C slightly overestimated by the model. We note, however, that the Sóller distribution is only representative of late summer – autumn season due to the coverage of this site being seasonally clustered around those seasons. This also suggests that our model also provides a realistic representation of seasonal characteristics.

Wind speed distributions are also similar between buoys and WRF, indicating that the model captures the typical range and frequency of wind speeds offshore. For wind direction, Palma, Sóller and Eivissa all show a dominant south-westerly sector and a secondary north-easterly contribution. WRF reproduces the main directional structure but shows a small counterclockwise shift toward slightly lower angles, with the most frequent directions displaced by about one bin (i.e., 15°). Part of these small differences may reflect measurement-height mismatches with the WRF diagnostic levels.

### Terrestrial evaluation using station observations

#### Data and preprocessing of land station observation

We evaluated the WRF-driven dataset **Balear1km** against observations from the Spanish National Meteorological Agency (AEMET) for the period 2009–2024. This observational network comprises 263 meteorological stations across the Balearic Islands measuring precipitation and daily maximum and minimum temperature, 47 of which also provide hourly data of temperature and precipitation. To ensure robustness, we retained only stations with at least ten years within 2009–2024, each containing a minimum of 90% of valid daily records. This resulted in 44 temperature stations and 149 precipitation stations for daily data. For the hourly analysis, we further required complete hourly coverage within valid days, reducing the hourly dataset to 18 temperature and 24 precipitation stations, still ensuring good spatial coverage over Mallorca, but sparse on the smaller islands.

Mean daily temperature was calculated as

1 $${T}_{{mean}}=\left({T}_{\max }+{T}_{\min }\right)/2$$

Where ${T}_{\max }$ and ${T}_{\min }$ are daily maximum and minimum temperatures, respectively. Observations and model data were compared at each station’s location using the nearest WRF grid cell over land. To ensure consistent sampling, every missing observational value was also set to missing in the corresponding WRF series. It should be noted, however, that while station data represent point measurements, WRF values correspond to grid-cell averages over an area of 1 km × 1 km, which may contribute to discrepancies.

#### Annual and spatial evaluation

**Fig. 5:**
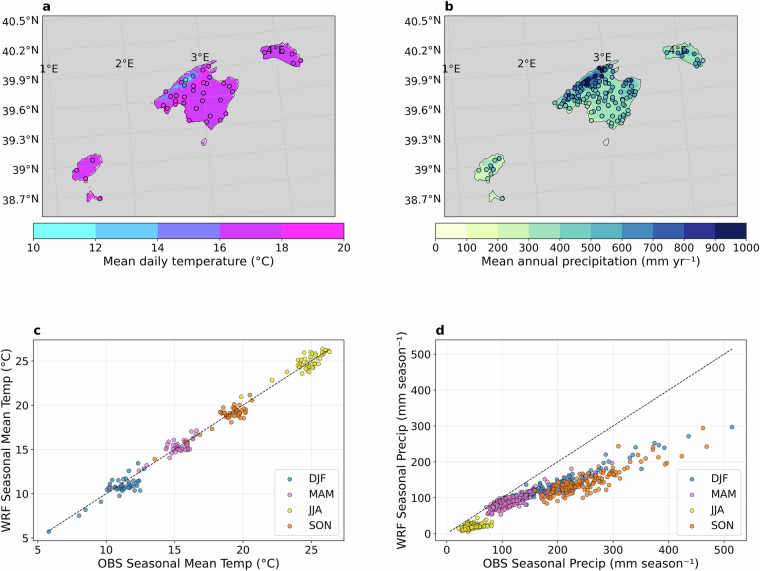
Spatial distribution of mean temperature (**a**) and annual precipitation (**b**) in WRF data (contours) and observations from AEMET Stations (markers) 2009–2024. Seasonal mean daily temperature (**c**) and seasonal precipitation (**d**) from WRF model vs Station observations (OBS). DJF is Decembre-January-February, MAM March-April-May, JJA June-July-August, SON September-October-November. Here only the overland portion of Balear1km is shown, since the comparison involves only land-based weather stations.

To assess the model’s overall performance, we first evaluate the spatial patterns of annual mean temperature and precipitation across the Balearic Islands (Fig. 5a,b). The WRF-driven dataset reproduces the observed distributions derived from AEMET station data, capturing the influence of topography and coastal gradients. Cooler and wetter conditions are correctly simulated over the elevated areas of Mallorca, particularly along the *Serra de Tramuntana* and the *Peninsula de Llevant* (Fig. 2), while lower-lying regions show relatively warmer and drier conditions.

The model’s temperature field shows a balanced distribution of biases across stations, with roughly half displaying a slight cold bias and the other half a warm bias (not shown). Simulated mean daily temperatures agree closely with observations, with station-level annual biases ranging from −1.3 °C to +1.1 °C and an average of −0.1 °C, showing that WRF reproduces temperature levels with high accuracy across the region.

In contrast, annual precipitation is systematically underestimated across all 149 stations (not shown). The mean bias amounts to −210.7 mm yr^−1^, corresponding to an average underestimation of −34.7%. Most of the strongest precipitation underestimations are observed at high-elevation sites, yet the largest single bias occurs at *Pollença Can Serra*, a foothill station facing the *Serra de Tramuntana*, where simulated rainfall totals are nearly 50% lower than observed. The mechanism behind the larger underestimations observed in mountainous regions is discussed in the next section.

Overall, WRF successfully reproduces the broad spatial gradients of temperature and precipitation but tends to underestimate total rainfall, particularly in complex terrain, which corresponds to the wettest areas of the Balearic Islands. This behavior is consistent with findings from a previous high-resolution regional simulation study in Western Mediterranean^35^.

#### Seasonal evaluation

The model also accurately reproduces the seasonal contrasts of temperature and precipitation (Fig. 5c-d). As expected, summer is the warmest and driest season, while spring is cooler and slightly wetter. Winter brings the coldest temperatures and substantial precipitation, with total rainfall amounts comparable to those in autumn. The seasonal precipitation totals are consistently underestimated by the model, except for six stations in spring where WRF produces marginally higher rainfall than observed. The model reproduces both the timing and the distinctive behavior of each season, showing that WRF realistically represents the regional seasonal variations.

**Fig. 6:**
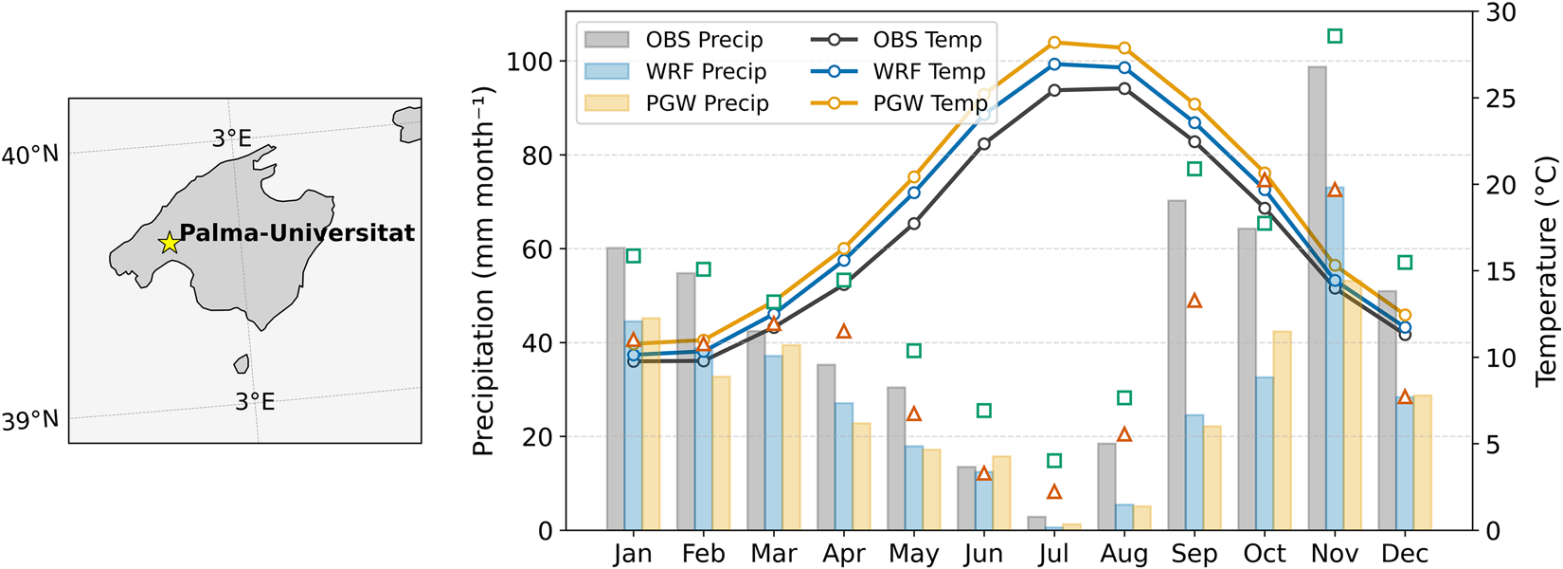
Climatograph of the station *Palma-Universitat* in 2009–2024. Mean temperature and accumulated precipitation for each calendar month from observations, WRF-CTL (WRF; historical) and WRF-PGW (PGW; pseudo-future). Green squares and red triangles show monthly precipitation values for Chelsa and WorldClim respectively.

At the local scale, the *Palma-Universitat* station provides an illustrative example of these seasonal characteristics (Fig. 6). The model reproduces the observed temperature cycle well, with a mean annual bias of about +1 °C and correct timing of seasonal extremes. Precipitation is also well phased, with maxima and minima in November and July, respectively, consistent with observations. However, rainfall amounts are underestimated during the wettest months, particularly in autumn, when cumulative precipitation is about 44% lower than observed during September-October-November. This local behavior mirrors the broader regional pattern, where precipitation deficits increase with total rainfall. The figure also includes the WRF-PGW simulation, representing how the 2009–2023 period would have behaved under a warmer climate. As this experiment describes a hypothetical future rather than an observed period, no direct validation is possible. Nonetheless, it provides valuable context for assessing potential climatic responses and shows the expected warming signal, consistent with projected global temperature increases.

**Fig. 7:**
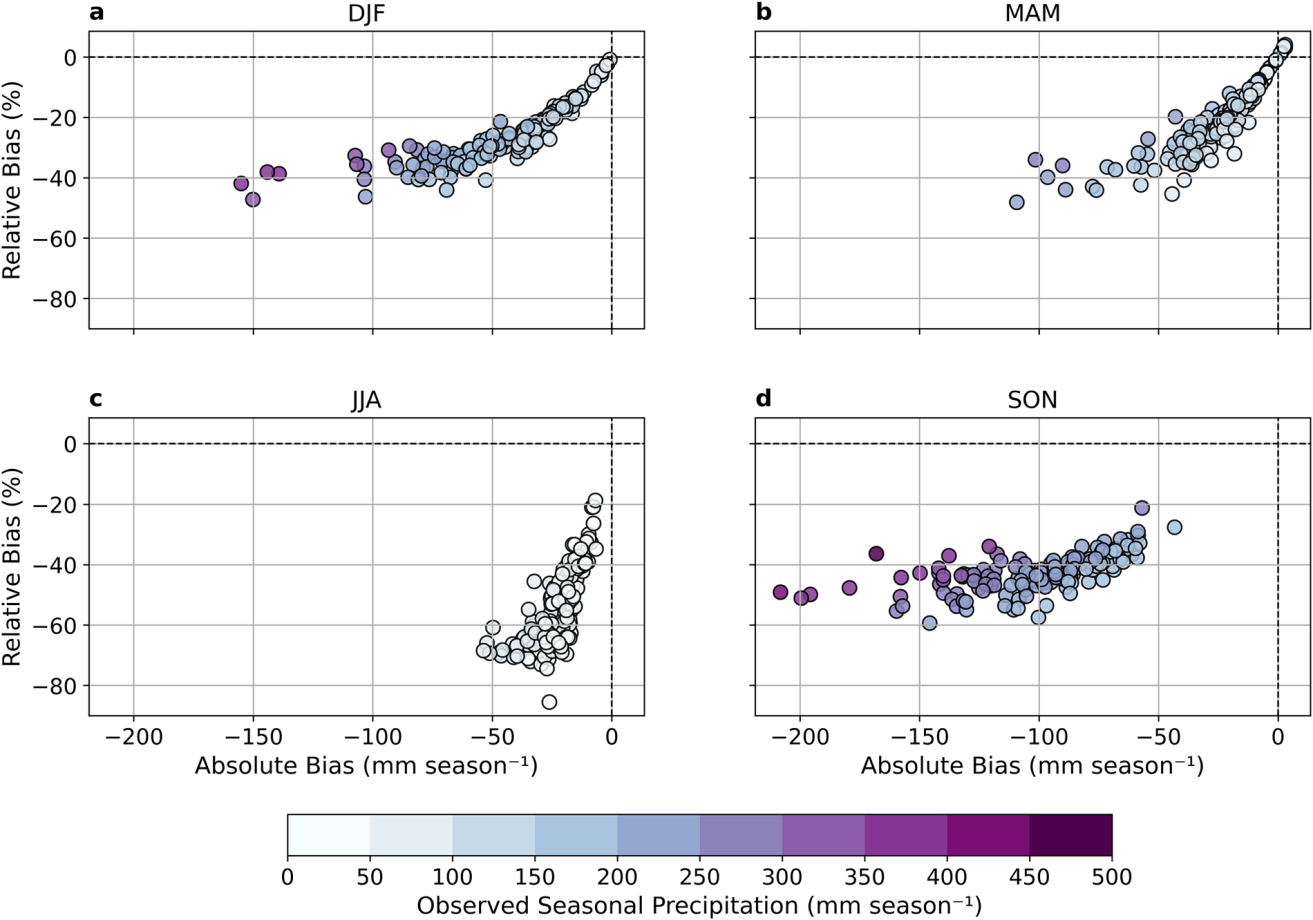
Scatter plots showing the relationship between relative and absolute seasonal precipitation biases at station locations for seasons December-January-February (DJF; **a**), March-April-May (MAM; **b**), June-July-August (JJA; **c**), September-October-November (SON; **d**). Color shading indicates the observed seasonal precipitation totals (mm season^−^¹) at each station.

Regionally, despite capturing the overall seasonal behavior, WRF systematically underestimates seasonal precipitation totals. Within each season, stations with higher precipitation totals generally show larger absolute underestimations (Figs. 5d, 7). This pattern reflects the event-based nature of rainfall, in contrast to temperature, which varies continuously. Because precipitation occurs in discrete episodes, the number of wet days differs notably between stations, causing cumulative underestimation to roughly scale with the frequency of wet events. Thus, winter and autumn, the wettest seasons, exhibit the largest station-level deficits, with individual stations bias reaching up to −47.2% (−150.2 mm) in winter and up to −59.4% (−145.7 mm) in autumn. By contrast, in summer, small seasonal totals make relative errors appear disproportionately large even for modest absolute differences, with a maximum underestimation of 85.5% at *Port d’Andratx - Es Rebolls* station, corresponding to only 26.1 mm of missing seasonal rainfall (Fig. 7).

WRF accurately reproduces the amplitude and timing of seasonal temperature cycle and captures the overall evolution of precipitation across seasons. However, total rainfall is almost always underestimated across all seasons, with the strongest biases occurring during the wettest stations-seasons. This is consistent with previous findings reporting a tendency toward underestimation in the regions of most intense rainfall in Southern France, showing convection-permitting simulations of extreme events in the Mediterranean still struggle to reproduce the most intense daily and hourly maxima (bias of about 200 mm day^−1^ and 40 mm h^−1^, respectively), despite notable improvement in the representation of spatial patterns, intensity, frequency, and variability compared to coarser-resolution simulations^35^.

#### Diurnal cycle and hourly evaluation

**Fig. 8:**
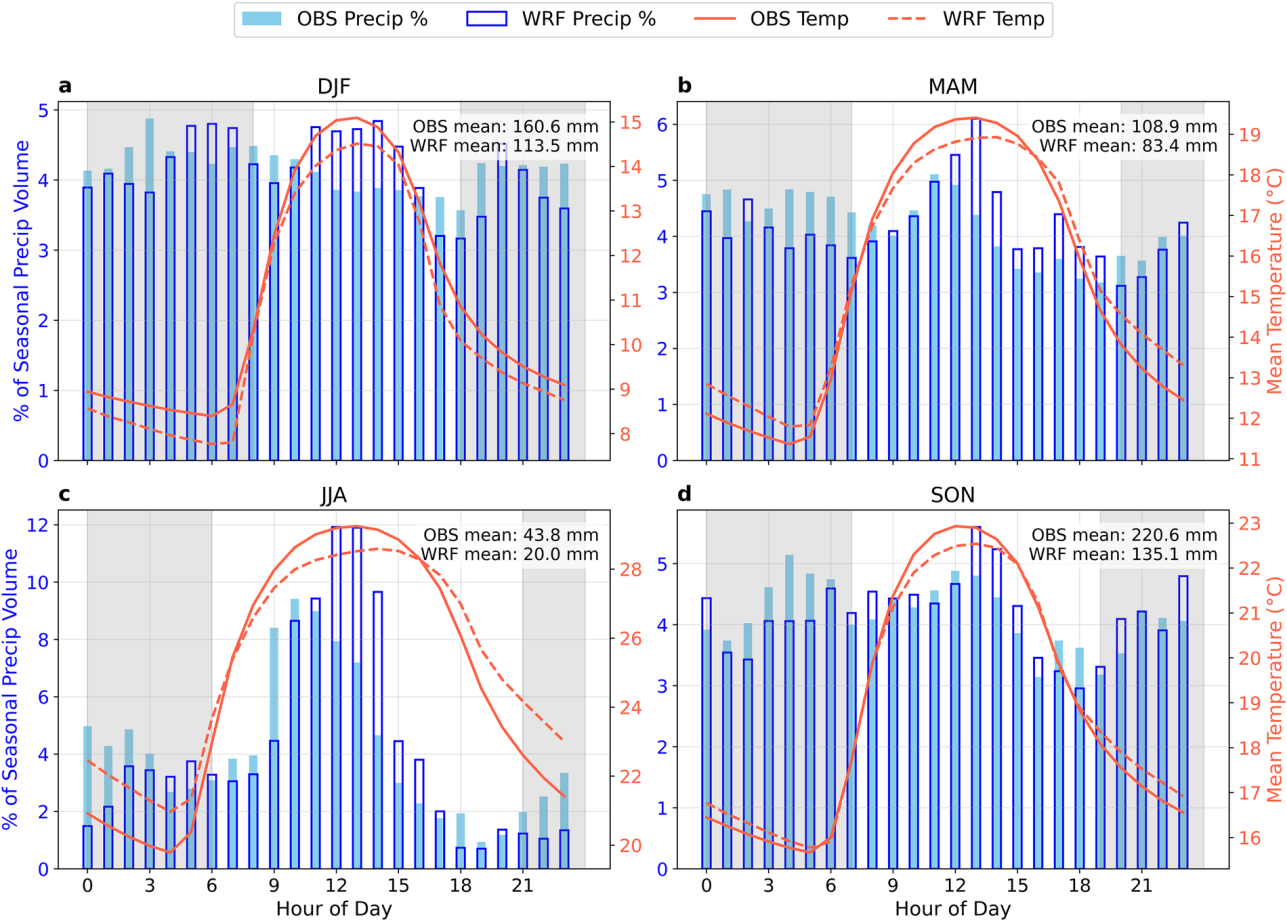
Seasonal diurnal cycles of mean temperature and hourly precipitation share for station-based observations and WRF simulations for seasons December-January-February (DJF; **a**), March-April-May (MAM; **b**), June-July-August (JJA; **c**), September-October-November (SON; **d**). Gray shades show approximate local nighttime for each season.

At hourly scale, the model reproduces temperature variations with high accuracy. Across the 18 stations with complete hourly records, the mean annual bias is +0.06 °C, and the root mean square error (RMSE) is 0.56 °C. The diurnal cycle is realistically captured, showing that WRF effectively represents both daytime warming and nocturnal cooling (Fig. 8). However, the midday temperature peak occurs slightly later than observed and is underestimated in all seasons.

The smallest mean daily temperature biases occur in spring and autumn, when warmer nights partly offset the daytime cold bias. In winter, the model remains too cold throughout the day, with an average bias of −0.5 °C. In contrast, summer shows insufficient nighttime cooling (up to +2 °C), producing a slight daily mean overestimation of about +0.5 °C. A slight delay in evening cooling is also apparent in spring and summer, while in winter the cooling starts earlier than observed.

In the warm season, precipitation exhibits a distinct diurnal cycle, clearly developed in summer when daytime convection dominates. In contrast, spring and autumn show only a weak trace of this signal, likely influenced by transitional months such as May and September. During winter, no diurnal cycle is evident in either the observations or the simulation. This seasonal contrast reflects the prevailing rainfall regimes, which are convective during the warm months and large-scale synoptic during the cold ones. WRF reproduces this general behavior, showing a summer diurnal signal consistent with observations but with a delayed rainfall peak. This delay in convective onset could be linked to the lower midday temperature peak, as reduced surface heating limits the buildup of near-surface instability that typically sets the stage for afternoon convection during summer. These biases may stem from model limitations in representing key physical processes, such as surface fluxes, cloud cover, soil moisture, or vertical mixing. Further exploration would be needed to identify the dominant mechanism, but the magnitude of these biases remains moderate and does not affect the overall consistency of the simulated climate.

Overall, WRF reproduces the phase of the diurnal cycles in temperature and precipitation realistically, as well as the seasonal regimes driving rainfall. The model thus represents both the seasonal characteristics and the diurnal behavior associated with typical weather patterns.

#### Extreme precipitation contribution to totals

Adequate representation of extreme precipitation is essential for impact assessment and hydrological applications. To evaluate how WRF captures such events, we define hourly extremes as those exceeding 13.8 mm h^−^¹, corresponding to the 99^th^ percentile of all wet-hour observations from 2009 to 2025 across the 19 stations with complete hourly records. These events represent only 1% of wet hours but account for a substantial share of total rainfall, particularly during the warm season.

**Fig. 9:**
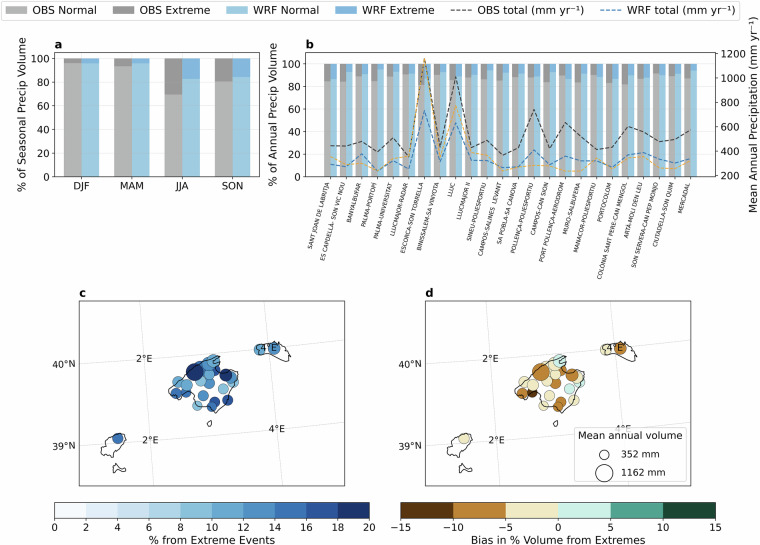
Contribution of hourly extremes (>13.8 mm h^−1^) to (**a**) total seasonal precipitation volume and (b) total annual precipitation volume at each station for observations and WRF. In (**b**), the yellow dashed line indicates the relative altitude of stations, which are ordered by longitude (west on the left, east on the right). Panels (**c,d**) show the spatial distribution of the contribution of hourly extremes to total annual precipitation for observations and the corresponding WRF–OBS bias, respectively.

The model reproduces the seasonal contribution of extreme events to total rainfall accurately (Fig. 9a). Summer shows the largest fractional contribution of hourly extremes to seasonal totals, followed by autumn, while winter and spring contribute less than 10%. This seasonal ranking aligns closely with observations and reflects the transition from convective dominance in summer to large-scale synoptic precipitation in winter. Across all stations, the total precipitation is generally underestimated, but the fraction of rainfall associated with extremes is realistically captured (Fig. 9b-d), indicating that WRF adequately simulates the contribution of most intense convective episodes to total rainfall.

A tendency of slight underestimation persists in the volume explained by extremes with a mean bias of percentage explained by extremes of only −3.8% across station (Fig. 9d), suggesting that the model marginally weakens the highest-intensity rainfall events contribution to totals. Nonetheless, more than 80% of the overall precipitation underestimation originates from non-extreme rainfall (not shown), implying that biases are mainly associated with moderate and light precipitation rather than with extremes. This result supports the suitability of the convection-permitting WRF configuration for studying the frequency and characteristics of intense precipitation over the Balearic Islands.

#### Summary of model performance

Overall, the WRF-generated dataset **Balear1km** provides a realistic representation of temperature and precipitation across the Balearic Islands. The model captures spatial and seasonal variability, including the influence of topography, the timing of seasonal cycles, and the structure of the diurnal variations. Temperature fields are well reproduced, showing negligible mean annual bias and realistic day-night amplitude. Precipitation is systematically underestimated, particularly in mountainous areas and during the wet seasons, though the relative contributions of heavy rainfall events are realistically represented. These results confirm that the dataset reliably describes the main climatic features of the region, although some limitations remain, most notably a general underestimation of precipitation that should be kept in mind when analyzing processes sensitive to rainfall. For applications requiring absolute rainfall amounts, such as hydrological modelling, bias-correction approaches can further enhance usability. However, for CTL-PGW change-signal analyses, it should be avoided or applied with caution, as it may modify the modeled response. Overall, Balear1km offers a robust and well-characterized basis for regional climate analysis and future impact studies.

### Contextualizing Balear1km precipitation using open high-resolution datasets

Station-based evaluation indicates that Balear1km precipitation is affected by a persistent underestimation, which is not surprising. Indeed, precipitation is widely recognized as one of the most challenging fields to reproduce in regional climate simulations and even more so in regions of complex terrain and high spatial gradients such as the Balearic Islands^37,56^. Thus, in this chapter we investigate further the place of Balear1km precipitation compared to reference datasets, which are the commonly used open-source high-resolution datasets CHELSA^29,57^ and WorldClim^28,58^. We investigate and discuss the benefits and limitations in the use of Balear1km for precipitations specifically.

**Fig. 10:**
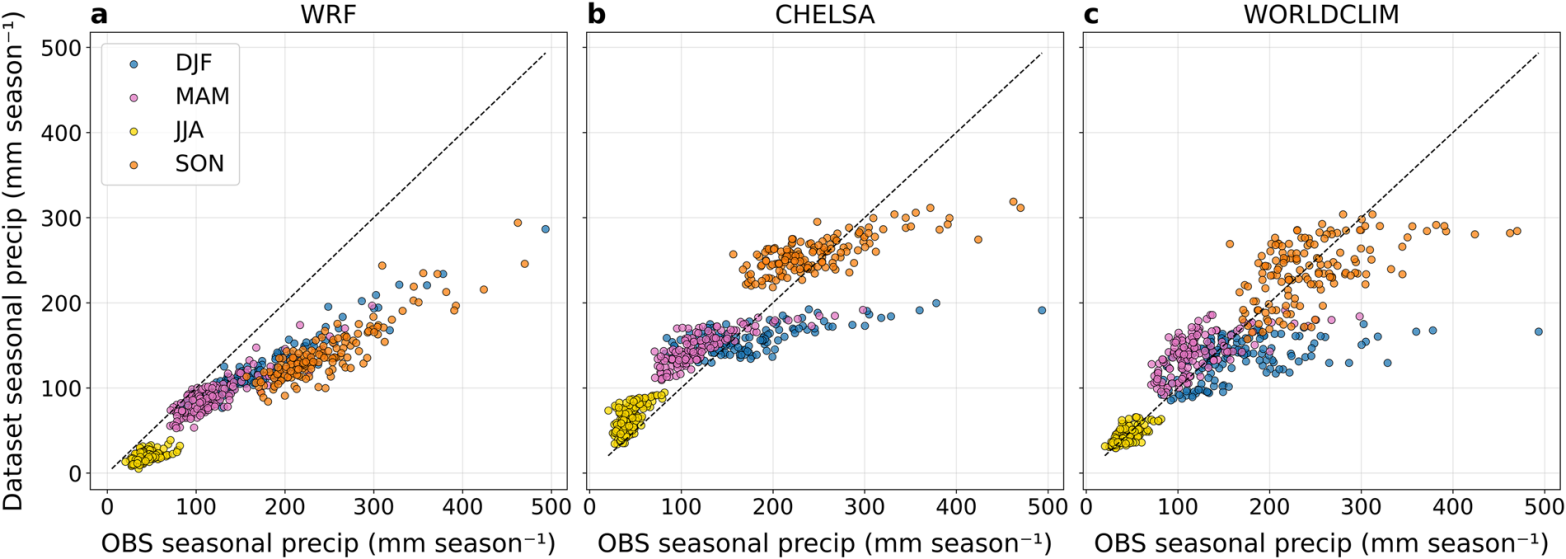
Mean seasonal precipitation of datasets WRF CTL (**a**), CHELSA (**b**), and WorldClim (**c**) vs Station observations (OBS).

**Fig. 11:**
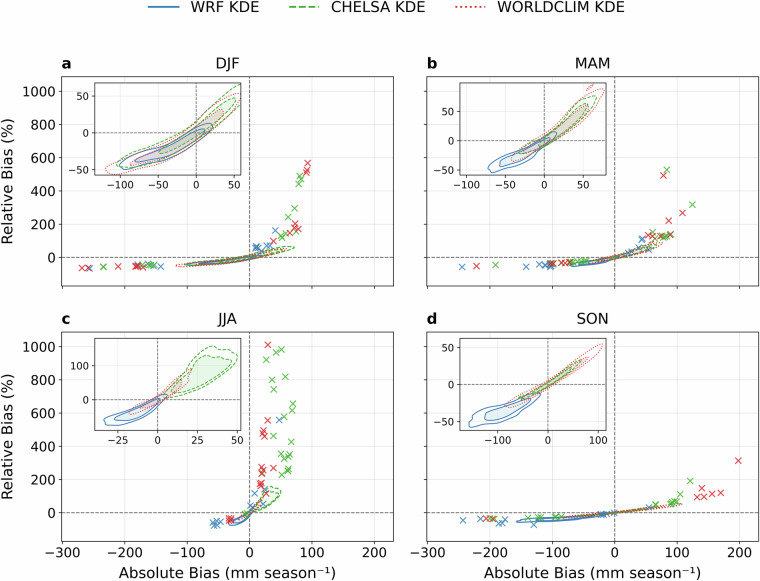
Kernel density estimates (KDE) of the joint distribution of relative and absolute seasonal precipitation biases with respect to station observations at station locations for WRF, CHELSA, and WorldClim, shown for December-January-February (DJF; **a**), March-April-May (MAM; **b**), June-July-August (JJA; **c**), and September-October-November (SON; **d**). Contours indicate the 70% and 90% highest-density regions, and inset panels zoom into the highest-density clusters of stations. Stations lying outside those main density clusters are shown as crosses.

Across all stations and seasons, WRF underestimates quasi-systematically seasonal precipitations, indicating a dominant, spatially coherent dry bias (Figs. 10, 11). In contrast, CHELSA and WorldClim station–season pairs span both overestimations and underestimations, except for CHELSA spring and summer precipitations which tend towards overestimation overall. Because station biases are centered around zero, the mean seasonal precipitation bias in CHELSA and WorldClim appears low. However, we note that their autumn and winter distribution appears comparatively flatter (i.e. a large station-to-station spread in observed seasonal precipitation, but a much smaller spread in the open-source reference datasets, Fig. 10), indicating weaker skill in representing seasonal precipitation spatial variability compared to WRF. This is consistent with greater station- and season-dependent variability in the biases, in contrast to the single dominant offset in WRF (Fig. 11). WRF, although biased low, better preserves spatial contrasts between drier and wetter stations, and its underestimation of winter and autumn precipitation is smaller at the wettest stations than in the reference datasets. Note that smaller mean biases in CHELSA and WorldClim are expected because both products are observation-constrained by construction. In contrast, **Balear1km** is dynamically modelled and, while it shows a systematic dry bias, its present-day precipitation magnitudes can be further improved through bias adjustment when required, without compromising its skill in representing spatial contrasts.

**Fig. 12:**
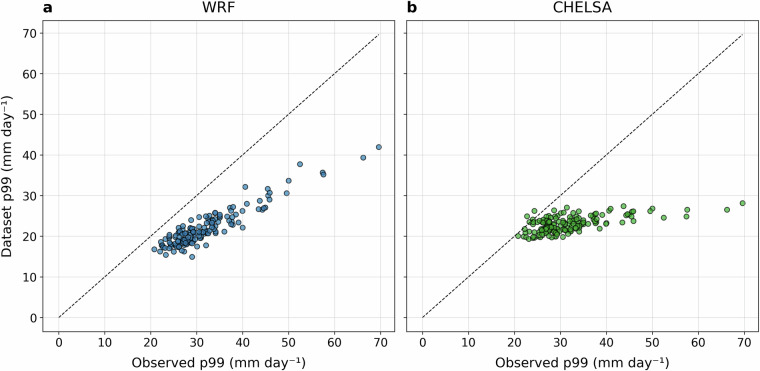
Scatter plots comparing station-based 99th percentiles of daily precipitation with dataset-derived values for WRF (**a**) and CHELSA (**b**). The dashed line indicates the 1:1 relationship. WorldClim is not included because it does not provide daily climatologies.

Given the high societal relevance of intense daily rainfall through its role in flooding, infrastructure impacts, agricultural losses, and risk management, we further assess precipitation performance in the upper tail of the daily distribution by comparing the 99th percentile at station locations for WRF and CHELSA, as WorldClim is only available at monthly scale. Similarly to the seasonal results, WRF underestimates daily extremes at all stations (Fig. 12), with a mean absolute bias of −9.92 mm and a mean relative bias of −30.3%. CHELSA shows a slightly smaller mean underestimation (mean absolute bias −8.89 mm; mean relative bias −25.1%), but a wider range of station-level errors, including occasional slight overestimation and larger negative outliers (down to −41.47 mm). Importantly, these larger departures occur for the wettest stations, indicating a reduced ability to capture the amplitude of the strongest extremes and a weaker station-to-station differentiation in the upper tail compared to WRF. Overall, Balear1km captures accurately where the strongest daily extremes tend to occur, while underestimating their magnitude, with an amplitude of underestimation comparable to CHELSA.

Finally, this comparison clarifies the complementarity and limitations of the open products for regional applications in the Balearic Islands region. WorldClim is only available at monthly resolution, constraining its use for daily variability and event-based analyses, including precipitation extremes, and both WorldClim and CHELSA are provided exclusively over-land. In contrast, **Balear1km** provides a physically consistent atmosphere over both land and the surrounding marine domain. Moreover, because **Balear1km** is produced with a dynamical regional model, projected changes are physically driven by the simulated circulation and thermodynamics interacting with the land-sea setting, resulting in a climate-change signal that is physically consistent across variables and across the full domain. While a precipitation bias adjustment may be required for applications focusing on present-days absolute amounts, **Balear1km** better captures spatial contrasts in both seasonal precipitation accumulations and daily precipitation extremes, and, although further application-specific validation may still be needed depending on the variables considered and the study objective, the extensive evaluation presented here indicates that Balear1km both improves the quality of high-resolution information currently available for the archipelago and substantially broadens the range of possible applications (physically coherent future projections, marine domain, multiple variables, and sub-daily analyses).

### Cross-domain usability checks

In addition to the evaluation against observations, we include three cross-domain demonstrations co-developed with local research teams. These demonstrations were designed and run with collaborators working in ecology, hydrology, and agroclimatology as a practical usability check. They confirm that the documented variables can be extracted and post-processed with consistent units and metadata, and with the expected spatial and temporal coverage, for use in ecology, hydrology, and agroclimatology.

**Fig. 13:**
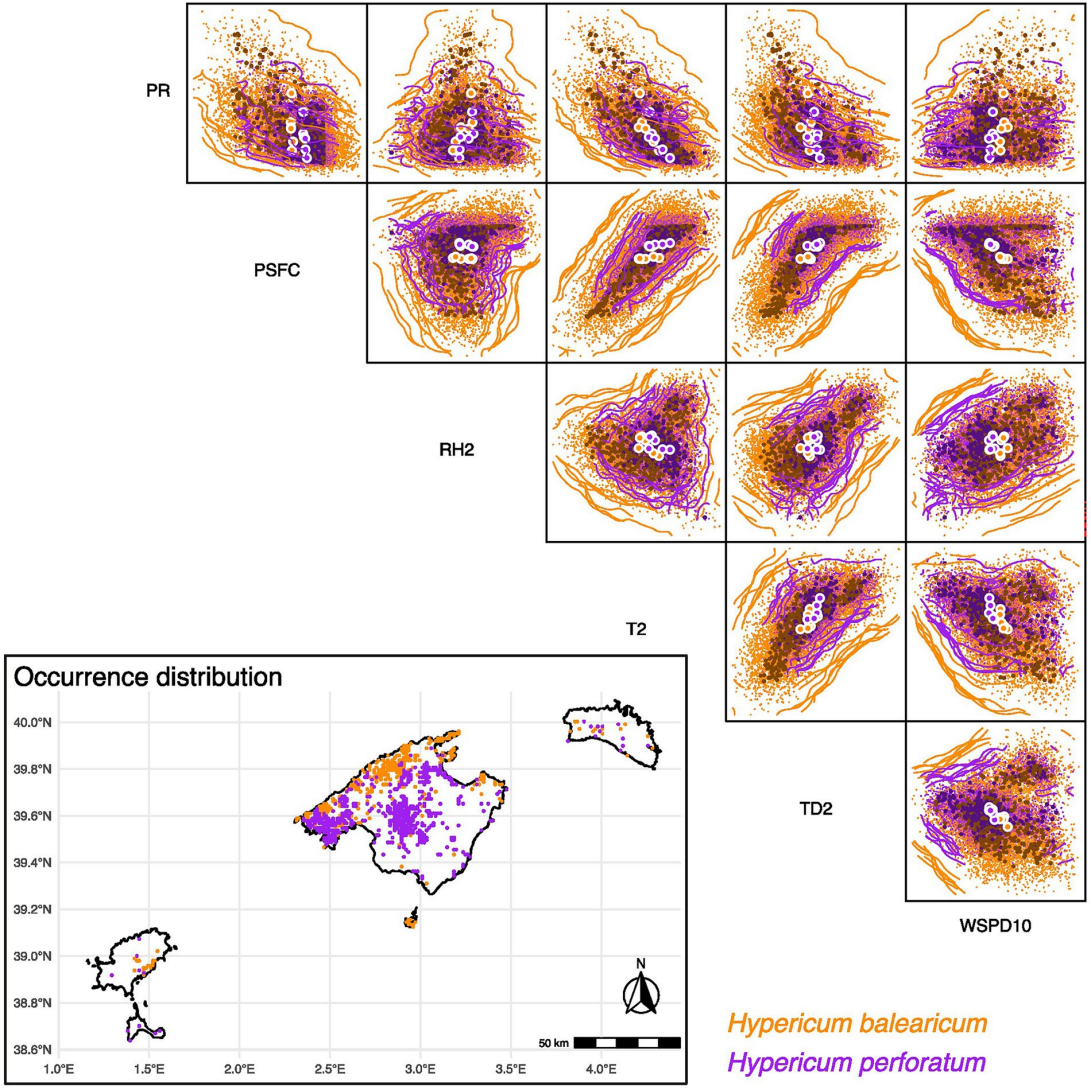
Six-dimensional environmental hypervolumes represent the climatic niches of *Hypericum balearicum* (orange) and *H. perforatum* (purple). Large points with white borders indicate the annual centroids of each species’ hypervolume. Small dark points show observed occurrence records, while small light points represent random samples drawn from the kernel density estimation within the defined bandwidth. Boundary lines are drawn to illustrate the extent of the niche in pairwise climatic dimensions. In the map, we reported the Balearic distribution of both species *H. balearicum* and *H. perforatum*. Occurrence data were downloaded from the Global Biodiversity Information Facility (GBIF; *H. balearicum*: GBIF.org (14 July 2025) 10.15468/dl.akxbyw; *H. perforatum*: GBIF.org (18 July 2025) 10.15468/dl.qzuxk2).

Ecology demonstration (Fig. 13). Occurrence records in the Balearic islands of two plant species, *Hypericum balearicum* and *H. perforatum*, were used to extract multi-year environmental data from the Balear1km variable dataset. These data were subsequently used to construct a multidimensional representation of each species’ environmental niche based on a hypervolume approach^59,60^. The figure shows the resulting hypervolume visualization and the associated occurrence mapping. These results indicate that, despite a narrow climatic overlap between the two species, in the Balearic archipelago, *H. perforatum* occupies a restricted subset of the broader environmental niche defined by *H. balearicum*.

**Fig. 14:**
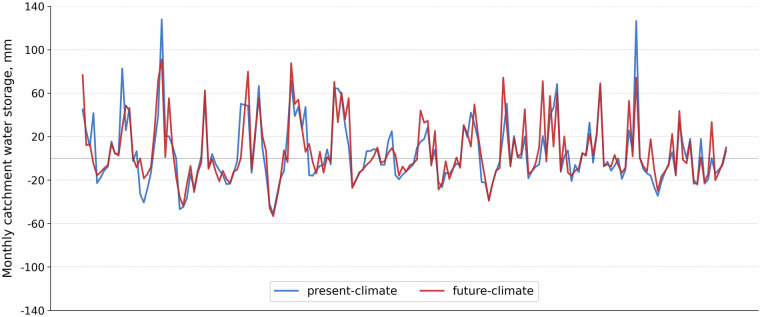
Evolution of the monthly catchment water storage in the *Albufera des Mercadal* (Menorca) for the present-climate and future-climate simulations for the period 2009–2023. The black line is zero value.

Hydrology demonstration (Fig. 14). Balear1km variables were aggregated over a defined catchment for the present-climate and pseudo-future simulations. A standard water-balance formulation^16,17^ using precipitation, evapotranspiration and runoff was then applied to derive a storage time series. The figure shows the resulting monthly storage series produced by this processing, showing that over the 180-month period rainfall totals were similar between simulations, while evapotranspiration decreased from 3,785 to 3,494.3 mm and total water storage increased from 787 to 1,078.4 mm, with runoff changing only slightly from 828.9 to 837.1 mm.

**Fig. 15:**
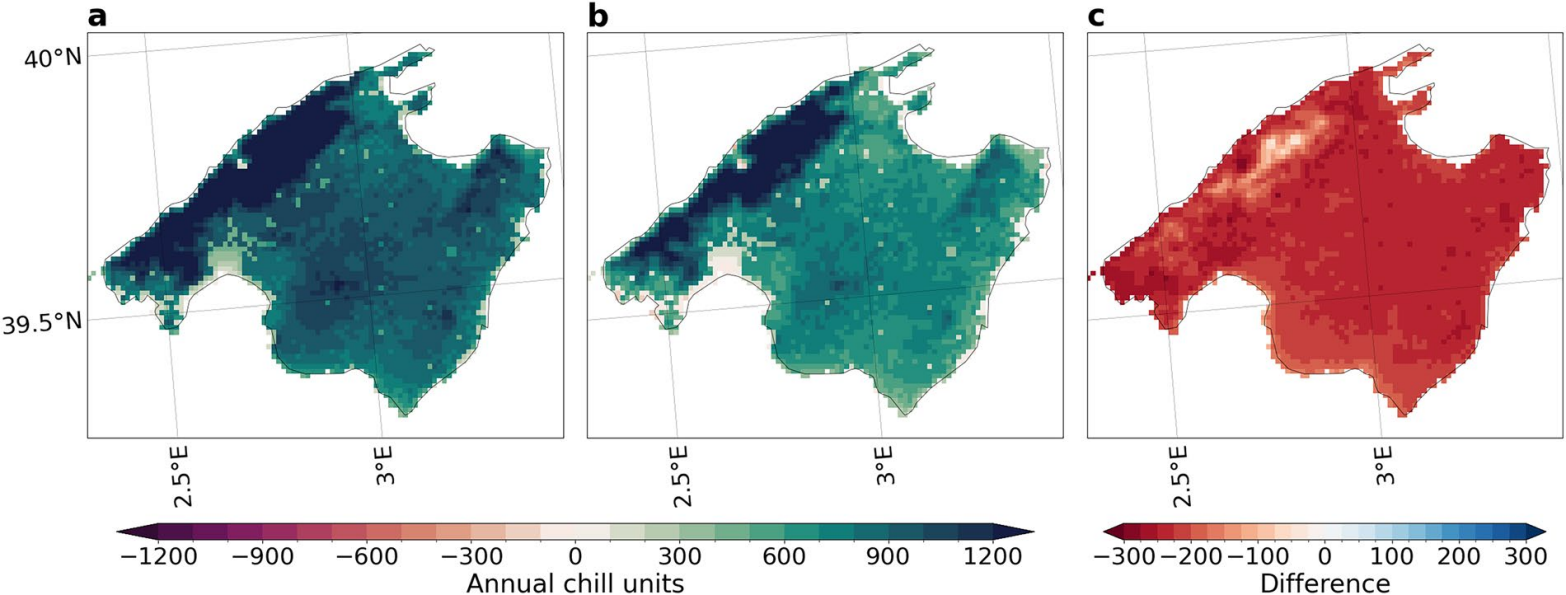
Present (**a**), future (**b**), and present-future difference (**c**) in the chilling units (CU; Utah model; Richardson *et al*., 1974) occurring in Mallorca (Balearic Islands) according to the Balear1km dataset.

Agroclimatic demonstration (Fig. 15). Hourly 2-m temperature was used to compute a winter chill metric using the Utah model^61^ over a fixed accumulation window. The figure shows the chill-unit (CU) fields for both experiments and their differences, as produced by this post-processing, showing that chill units decrease consistently across Mallorca by 25.2 to 293.6 CU depending on location, and that the area accumulating less than 600 CU (i.e., making sweet cherry and many traditional almond tree varieties non-viable) increases from 10.3% to 30.3% and the area accumulating less than 300 CU (i.e., making most fruit trees non-viable) increases from 3.8% to 8.5% in the pseudo-future simulation, using these two illustrative thresholds, which are commonly reported for almond cultivar chill requirements^62–64^.

## Usage Notes

### Understanding the scope and limitations of the dataset for interpretation

Regional climate simulations based on dynamical downscaling serve different purposes depending on their boundary forcing. When driven by reanalysis data, as in this study, the regional model remains constrained by the observed large-scale circulation, reproducing the actual sequence of past atmospheric events within the regional domain. This contrasts with simulations downscaled from Global Climate Models (GCMs), as GCMs are designed to represent the statistical behavior of the climate system rather than the exact chronology of events. Indeed, GCMs evolve freely according to their internal variability and are constrained only by external forcings such as solar radiation, greenhouse gas concentrations, aerosols, and prescribed land-use or emission scenarios. In the Pseudo-Global Warming (PGW) framework used here, the reanalysis fields are retained as boundary conditions but are modified to include the large-scale climate-change signal derived from GCM ensembles. This preserves the observed chronology and synoptic evolution of weather systems during 2009–2024 while embedding the perturbations associated with climate change, thereby allowing an assessment of how known events and regional processes might respond under future climate conditions.

It is especially important that users of pseudo-future data created with a PGW approach understand its construction and limitations. The PGW method is not a formal climate projection and does not simulate specific future years. Instead, it models how recent historical years would have behaved in an atmosphere altered by future-climate thermodynamic characteristics (e.g. warmer temperatures, increased moisture). This approach introduces limitations that must be considered when interpreting results.

The initial and boundary conditions for the PGW simulation (i.e., pseudo-future) are derived from the reanalysis fields of 2009–2023, meaning that the same sequence of large-scale weather systems enters the model domain as in the historical period. The experiment therefore does not show whether such systems would occur in a future scenario, but rather indicates how those historical systems would evolve under future conditions in the simulated region. Thus, while PGW simulations incorporate thermodynamic changes associated with warming, such as higher temperatures and greater atmospheric moisture, they only partially represent dynamical changes. The large-scale atmospheric configuration is largely preserved from the reanalysis, with only modest adjustments introduced through the imposed monthly climate-change signal. As a result, PGW simulations allow for the examination of how the structure, intensity, or evolution of weather systems may respond to a warmer climate. However, users should be cautious when interpreting potential changes in their frequency. Similarly, interpretations of geographic occurrence should be made with care, particularly for weather systems governed by large-scale dynamical processes, such as the winter mesoscale cyclonic systems characteristic of our region.

Thus, PGW results should not be interpreted as a prediction of future weather but as one plausible evolution of familiar weather systems in a different climate context. To avoid misunderstanding or overstatement, users of pseudo-future data are advised to avoid language that implies determinism, such as “will happen” or “projection”. Instead, more appropriate vocabulary includes terms like “simulated response under future-like conditions”, “plausible future scenario”, or “climate-perturbed analogue”. This helps clarify the methodological intent of PGW experiments: to explore how past weather systems would behave under the thermodynamic conditions associated with climate change, without implying that those systems or their outcomes will necessarily occur in the future.

### WRF simulations static geographic information file and use

In addition to the simulation outputs, we provide the *geo_em.d02.nc* file in the Supplementary. This file is generated by the geogrid module of the WRF Preprocessing System (WPS), which defines the model domain and supplies the geographical fields required to run WRF. It contains high-resolution (1 km) static inputs for the 320 × 220 child domain, including topography, land use, vegetation, albedo, soil type, and landmask, along with the Lambert conformal projection and coordinate definitions used by the model.

## Supplementary information

Supplementary Information

Supplementary Files

Supplementary Files

## Data Availability

Further details on the **Balear1km** dataset^55^ content e *Data Record* section. All data generated and analyzed in this study are openly available in the University of the Balearic Islands Dataverse repository, maintained by the Consortium of University Services of Catalonia (CSUC). The dataset, titled “*Dynamically downscaled high-resolution (1 km) climate information over the Balearic Islands*” can be accessed at https://dataverse.csuc.cat/dataset.xhtml?persistentId = doi:10.34810/data2071. The collection contains 1,260 CF-compliant NetCDF files providing hourly atmospheric variables over the Balearic Islands for two experiments: a historical Control simulation (2009–2024) and a Pseudo-Global Warming experiment including projected climate change effects. Data are organized by variable and experiment (Fig. 3) with each monthly file representing a single variable—precipitation rate, surface pressure, relative humidity, air temperature, dewpoint temperature, or wind speed—on a 1 km-resolution grid. The dataset can be located by filtering the UIB Dataverse by year (2025) and subject area (Physics or Earth and Environmental Sciences).

## Code availability

In the Supplementary Material, we provide the WRF and WPS *namelist* files used to generate the simulations to facilitate reproducibility.

We used the code PGWERA5WRF^65^ (v1.1), publicly available on Zenodo (10.5281/zenodo.10555108) to compute the climate change signal and create boundary conditions for the Pseudo-future WRF simulation. The code provides tools to combine ERA5 reanalysis with CMIP6 multi-model anomalies to build boundary conditions for Pseudo-Global Warming (PGW) experiments with WRF. The workflow includes scripts for data preprocessing, interpolation to ERA5 grids and pressure levels, and generation of WRF-intermediate files including the PGW signal.
